# Mathematical formulation and computation of the dynamics of blood flow, heat and mass transfer during MRI scanning

**DOI:** 10.1038/s41598-024-56844-2

**Published:** 2024-03-16

**Authors:** Annord Mwapinga

**Affiliations:** Mwenge Catholic University: Department of Natural Sciences and Information Technology, P.O. Box 1226, Moshi, Tanzania

**Keywords:** Magnetic resonance imaging, Stenosis, Chemical reaction Joule heating, Computational biology and bioinformatics, Mathematics and computing, Physics

## Abstract

Computational modeling of arterial blood flow, heat and mass transfer during MRI scanning is studied. The flow is assumed to be unsteady, in-compressible, and asymmetric. Mathematical formulation considers the presence of stenosis, joule heating viscous dissipation and chemical reaction. The explicit finite difference scheme is used to numerically solve the model equations. The MATLAB software was used to plot the graphical results. The study reveals that, during MRI scanning, both radial and axial velocities diminish with increase in the strength of magnetic fields. Besides, the study found that, Eckert number and Hartman number enhance the blood’s temperature and the same, diminishes with increase in Prandtl and Reynolds numbers. Concentration profile is observed to decline with increase in chemical reaction parameter, Schmidt number and Reynolds number. Soret number on the other hand, is observed to positively influence the concentration.

## Introduction

One of the radiology techniques in hospitals is the use of magnetic resonance imaging (MRI). MRI is currently, the clinically favorite method for imaging soft tissue as it can produce a purer, more detailed view of internal organs than computed tomography (CT). MRI uses no radiation and delivers superb images of the body with no recognized harmful health effects. Blood contains iron in red blood cells. The presence of iron in blood has drawn much attention to mathematicians to determine the dynamics of blood flow when the body is subjected to the magnetic fields. In that regard, blood possesses electrically conducting and magnetization behavior because of the ions which are present in the plasma. Blood is a transporting agent in the human body. gases such as oxygen and hydrogen are transported via blood in human body. If blood abnormally flow, it affects the transportation of several materials in the body, this includes heat and oxygen. According to^1^, blood is a nutrient- and oxygen-rich bodily fluid that facilitates the body’s natural disposal of metabolic byproducts. The blood in the artery travels to all parts of the body, making it a crucial part of sustaining life.

Besides, human arterial wall may consists of fat deposits (plaques or stenosis) that reduce the radius of the artery and hence disturbing the normal flow of blood. Several scholars have modeled blood flow in along an ill-artery. This includes the study by^2–5^ and^6^.

The similarity and finite difference solution on biomagnetic flow and heat transfer of Blood $-Fe_3O_4$ through a thin needle was studied by^7^. The study assumed blood to be base fluid which exhibits electrical conductivity and polarization properties. A similar study was done by^8^ where the fluid transport behavior of the flow of gold (Au)-copper (Cu)/biomagnetic blood hybrid nanofluid in an inclined irregular stenosis artery as a consequence of varying viscosity and Lorentz force was addressed. Besides,^9^ carried out a fractional modeling of non-Newtonian Casson fluid squeezed between two parallel plates. The study was performed under the influence of magneto-hydrodynamic and Darcian effects.

On the other hand, a mathematical model of non-Newtonian blood flow, heat and mass transfer through a stenosed artery wa studied by^10^. The Herschel-Bulkley model was chosen to suit the non-Newtonian characteristics. Body acceleration, magnetic fields and chemical reaction were taken into consideration. Some other aspects of heat transfer was similarly studied by^11^. However the study involved natural convection in nanofluid flow with chemotaxis process over a vertically inclined heated surface. Furthermore, similar study was carried out as shown by^12–16^ and^17^.

Several scholars have studied MHD blood flow in arteries, this include^18–21^, and^22^. Besides,^23^ investigated the effect of magnetic fields when a human body is subjected to body accelerations and chemical reactions. This was a special case of the magnetic therapy. The finite difference method was used to solve the resulting partial differential equations.

The main purpose of the current study is to investigate the dynamics of blood flow, heat and mass transfer during MRI scanning. The constriction of the artery, Joule heating, and chemical reactions are considered. The findings of the current investigation are very useful in clinical rheology departments, more important, the findings can be used to control fluid flow. The governing model equations involved are the momentum, energy and the concentration. To these flow equations, some assumptions as stipulated in formulation part are applied to come up with some desired model equations. The model equations are solved numerically using finite difference schemes. The MATLAB software is used to produce graphical results.

## Mathematical formulation

**Figure 1:**
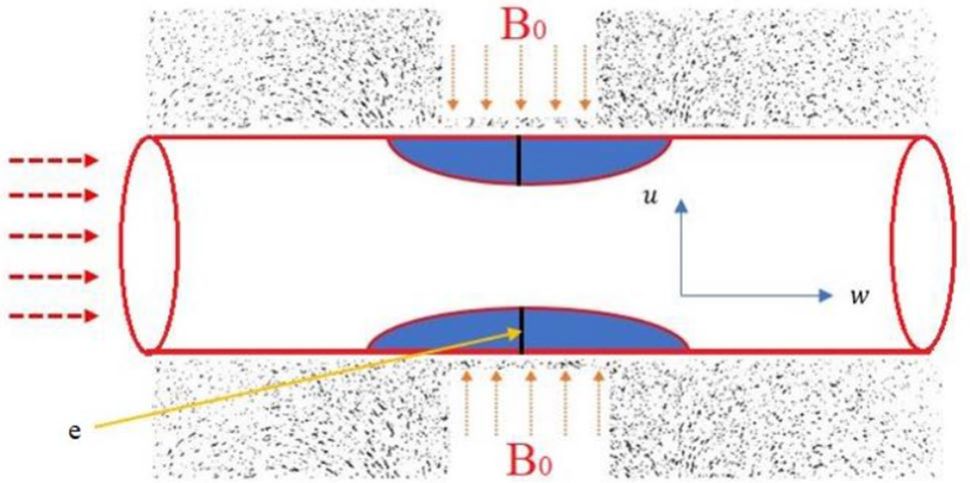
The schematic diagram of the constricted artery.

The current study considers that, the flow is unsteady, laminar, in-compressible, asymmetric, fully developed, and horizontal. MRI scanning machines produces magnetic fields that are perpendicular to the axis of symmetry, Joule or ohmic heating is in place, the artery is cylindrical, there is no an external heat source. Furthermore, the electrical conductivity, thermal conductivity and blood’s viscosity are constant. It is also considered that $r=0$ is the axis of the asymmetric flow and $u_r, u_\theta , u_z$ are the velocities in ($r, \theta , z$) directions. Under these assumptions therefore, the velocity is independent of angle $\theta$ that is $\dfrac{\partial }{\partial \theta }=0$. Blood is considered to be a chemically reacting fluid. In Figure 1, a Schematic diagram diagram of a stenosed artery is shown.

The study assumes further that along radial direction, the pressure gradient is negligible because the lumen radius of the artery is small. However, the pressure gradient along axial direction is given by

2.1 $$  - \frac{{\partial P}}{{\partial z}} = A_{0}  + A_{1} \cos (nt) $$

where $A_0$ is a steady state part of pressure gradient and $A_1$ is the amplitude of oscillatory. $n=2\pi f$ with *f* being the heart pulse frequency and *t* is the time. The constricting part of the artery is defined as

$$\begin{aligned} H(z)= {\left\{ \begin{array}{ll} r_0 - e &{} \left[ 1+\cos \left( \dfrac{\pi z}{2z_0}\right) \right] \hbox { if } -2z_0\le z \le 2z_0 \\ r_0 &{} \hbox { Otherwise } \end{array}\right. } \end{aligned}$$

Where $r_0$ is the radius of the normal artery, *H*(*z*) is the radius of the reduced artery and $\delta$ is the height of stenosis. Following^24^, the controlling blood flow model equals becomes;

2.2 $$\begin{aligned}&\dfrac{\partial u}{\partial r}+\dfrac{u}{r}+\dfrac{\partial w}{\partial z}=0 \end{aligned}$$

2.3 $$\begin{aligned}{}&\rho \left( \dfrac{\partial u}{\partial t}+u\dfrac{\partial u}{\partial r}+w\dfrac{\partial u}{\partial z}\right) =\mu \left( \dfrac{\partial ^2 u}{\partial r^2}+\dfrac{1}{r}\dfrac{\partial u}{\partial r}-\dfrac{u}{r^2}+\dfrac{\partial ^2 u}{\partial z^2} \right) \nonumber \\&\rho \left( \dfrac{\partial w}{\partial t}+u\dfrac{\partial w}{\partial r}+w\dfrac{\partial w}{\partial z}\right) =A_{0}+A_{1}\cos (nt)+\mu \left( \dfrac{\partial ^2 w}{\partial r^2}+\dfrac{1}{r}\dfrac{\partial w}{\partial r}+\dfrac{\partial ^2 w}{\partial z^2} \right) -\sigma {B^2_{0}}w \end{aligned}$$

2.4 $$\begin{aligned}&\rho C_p\left( \dfrac{\partial T}{\partial t}+u\dfrac{\partial T}{\partial r}+w\dfrac{\partial T}{\partial z}\right) =k\left( \dfrac{\partial ^2 T}{\partial r^2}+\dfrac{1}{r}\dfrac{\partial T}{\partial r}+\dfrac{\partial ^2 T}{\partial z^2} \right) + \phi +\sigma {B^2_{0}}w^2 \end{aligned}$$

2.5 $$\begin{aligned}&\dfrac{\partial C}{\partial t}+u\dfrac{\partial C}{\partial r}+w\dfrac{\partial C}{\partial z}=D\left( \dfrac{\partial ^2 C}{\partial r^2}+\dfrac{1}{r}\dfrac{\partial C}{\partial r}+\dfrac{\partial ^2 C}{\partial z^2} \right) + \dfrac{DK_T}{T_w}\left( \dfrac{\partial ^2 T}{\partial r^2}+\dfrac{1}{r}\dfrac{\partial T}{\partial r}+\dfrac{\partial ^2 T}{\partial z^2} \right) -\beta C \end{aligned}$$

where the function $\phi$ is the viscous dissipation given by

2.6 $$\begin{aligned} \phi =\mu \left[ \left( \dfrac{\partial u}{\partial r}+\dfrac{\partial v}{\partial z}\right) ^2+2\left( \dfrac{\partial v}{\partial r}\right) ^2+2\left( \dfrac{\partial u}{\partial z}\right) ^2 \right] \end{aligned}$$

### Scaling the variables

This sub-section the non-dimensional variables are introduced. The characteristic fluid velocity $W_{c}$ and distance $r_{c}$ are used. The $W_{c}$ is assumed to be the average blood’s velocity flowing along the artery. Similar approach was used by^25,26^ and^27^.

2.7 $$\begin{aligned}&\eta = \dfrac{r}{r_{c}},\quad z^*=\dfrac{z}{r_{c}},\quad w^*= \dfrac{w}{W_{c}},\quad u^*= \dfrac{u}{W_{c}},\quad \tau =\dfrac{tW_{c}}{r_{c}},\quad A_{0}^*=\dfrac{A_{0}r_{c}}{\rho W^2_{c}}\quad \end{aligned}$$

2.8 $$\begin{aligned}&A_{1}^*=\dfrac{A_{1}r_{c}}{\rho W^2_{c}},\quad m=\dfrac{r_{c}n}{W_{c}}, \quad T^*=\dfrac{T-T_0}{T_w-T_0}, \quad \quad C^*=\dfrac{C-C_0}{C_w-C_0}, \quad \beta ^*=\dfrac{\beta r^2_c}{\nu } \end{aligned}$$

Using the non-dimensional variables above and dropping the asterisks, we get the following blood flow model equations

2.9 $$\begin{aligned} \dfrac{\partial u}{\partial \eta }+\dfrac{u}{\eta }+\dfrac{\partial w}{\partial z}=&0 \end{aligned}$$

2.10 $$\begin{aligned} \dfrac{\partial u}{\partial \tau }+u\dfrac{\partial u}{\partial \eta }+w\dfrac{\partial u}{\partial z} =&\dfrac{1}{\text {Re}} \left( \dfrac{\partial ^2 u}{\partial \eta ^2}+\dfrac{1}{\eta }\dfrac{\partial u}{\partial \eta }-\dfrac{u}{\eta ^2}+\dfrac{\partial ^2 u}{\partial z^2} \right) \end{aligned}$$

2.11 $$\begin{aligned} \dfrac{\partial w}{\partial \tau }+u\dfrac{\partial w}{\partial \eta }+w\dfrac{\partial w}{\partial z} =&A_{0}+A_{1}\cos (mt)+\dfrac{1}{\text {Re}} \left( \dfrac{\partial ^2 w}{\partial \eta ^2}+\dfrac{1}{\eta }\dfrac{\partial w}{\partial \eta }+\dfrac{\partial ^2 w}{\partial z^2} \right) -\dfrac{\text {M}^2}{\text {Re}}w \end{aligned}$$

2.12 $$ \begin{aligned}   \frac{{\partial T}}{{\partial \tau }} + u\frac{{\partial T}}{{\partial \eta }} + w\frac{{\partial T}}{{\partial z}} =  & \frac{{\text{1}}}{{{\text{Pr Re}}}}\left( {\frac{{\partial ^{2} T}}{{\partial \eta ^{2} }} + \frac{1}{\eta }\frac{{\partial T}}{{\partial \eta }} + \frac{{\partial ^{2} T}}{{\partial z^{2} }}} \right) + \frac{{{\text{Ec}}}}{{{\text{Re}}}}{\text{M}}^{2} w^{2}  \\     &  + \frac{{{\text{Ec}}}}{{{\text{Re}}}}\left[ {2\left( {\frac{{\partial w}}{{\partial \eta }}} \right)^{2}  + 2\left( {\frac{{\partial u}}{{\partial z}}} \right)^{2}  + \left( {\frac{{\partial w}}{{\partial z}} + \frac{{\partial u}}{{\partial \eta }}} \right)^{2} } \right] \\  \end{aligned}  $$

2.13 $$ \begin{aligned}   \frac{{\partial C}}{{\partial \tau }} + u\frac{{\partial C}}{{\partial \eta }} + w\frac{{\partial C}}{{\partial z}} =  & \frac{1}{{{\text{Sc Re}}}}\left( {\frac{{\partial ^{2} C}}{{\partial \eta ^{2} }} + \frac{1}{\eta }\frac{{\partial C}}{{\partial \eta }} + \frac{{\partial ^{2} C}}{{\partial z^{2} }}} \right) + \frac{{{\text{Sr}}}}{{{\text{Re}}}}\left( {\frac{{\partial ^{2} T}}{{\partial \eta ^{2} }} + \frac{1}{\eta }\frac{{\partial T}}{{\partial \eta }} + \frac{{\partial ^{2} T}}{{\partial z^{2} }}} \right) \\     &  - \frac{1}{{{\text{Re}}}}\beta C \\  \end{aligned}  $$

where $\text {Re}=\dfrac{\rho W_c r_c}{\mu }$, $\text {M}=B_0 W_c\sqrt{\dfrac{\sigma }{\mu }}$, $\text {Pr}=\dfrac{\mu c_p}{k}$, $\text {Ec}=\dfrac{W^2_c}{c_p (T_w-T_0)}$, $\text {Sc}=\dfrac{\nu }{D}$ and $\text {Sr }=\dfrac{DK_T(T_w-T_0)}{\nu (C_w-C_0)}$ are respectively Reynolds number, Hartman number, Prandtl number, Eckert number, Schmidt number and Soret number.

The boundary and initial conditions in dimensionless form become;

2.14 $$\begin{aligned}&w(\eta ,z,0)=w_{0},\quad T(\eta ,z,0)=T_{0}, \quad C(\eta ,z,0)=C_{0}\quad \quad \quad \quad \quad \quad \quad \quad \quad \end{aligned}$$

2.15 $$\begin{aligned}&w(\eta ,z,t)=u(\eta ,z,t)=0, T(\eta ,z,t)=T_{w}, C(\eta ,z,t)=C_{w}\quad \text {on}\quad \eta =H(z) \end{aligned}$$

2.16 $$\begin{aligned}&\dfrac{\partial w(\eta ,z,t)}{\partial \eta }=\quad \dfrac{\partial T(\eta ,z,t)}{\partial \eta }=\dfrac{\partial C(\eta ,z,t)}{\partial \eta }= u(\eta ,z,t)=0, \text {on}\quad \eta =0 \end{aligned}$$

## Solution of the problem

### Radial coordinate transformation

In this section we find the solution of the formulated model. The finite difference method is used. However, before dicretizing, we minimize chances of interpolation errors by introducing the radial coordination transformation $\xi =\dfrac{\eta }{H(z)}$. This aims at immobilizing the constricted artery into a rectangular domain. Using such suitable transformation, the continuity, momentum, energy and mass transfer equations becomes;

3.1 $$\begin{aligned}&\dfrac{1}{H}\dfrac{\partial u}{\partial \xi }+\dfrac{u}{H\xi }+\dfrac{\partial w}{\partial z}-\dfrac{\xi }{H}\dfrac{d H}{d z}\dfrac{\partial w}{\partial \xi }=0 \end{aligned}$$

3.2 $$\begin{aligned} \dfrac{\partial u}{\partial \tau }=&-\dfrac{u\partial u}{H\partial \xi }-w\left( \dfrac{\partial u}{\partial z}-\dfrac{\xi }{H}\dfrac{dH}{dz}\dfrac{\partial u}{\partial \xi }\right) +\dfrac{1}{\text {Re} H^2} \left( \dfrac{\partial ^2 u}{\partial \xi ^2}+\dfrac{1}{\xi }\dfrac{\partial u}{\partial \xi }-\dfrac{u}{\xi ^2} \right) \nonumber \\&+\dfrac{1}{\text {Re}}\left[ \dfrac{\partial ^2 u}{\partial z^2}-\dfrac{2\xi }{H} \dfrac{dH}{dz}\dfrac{\partial ^2 u}{\partial \xi \partial z}-\dfrac{\xi }{H}\dfrac{d^2 H}{dz^2}\dfrac{\partial u}{\partial \xi }+\dfrac{\xi ^2}{H^2}\left( \dfrac{dH}{dz} \right) ^2\dfrac{\partial ^2 u}{\partial \xi ^2}+\dfrac{3\xi }{H^2} \left( \dfrac{dH}{dz} \right) ^2 \dfrac{\partial u}{\partial \xi } \right] \end{aligned}$$

3.3 $$\begin{aligned} \dfrac{\partial w}{\partial \tau }=&-\dfrac{u\partial w}{H\partial \xi }-w\left( \dfrac{\partial w}{\partial z}-\dfrac{\xi }{H}\dfrac{dH}{dz}\dfrac{\partial w}{\partial \xi }\right) +(A_{0}+A_{1}\cos (m\tau ))+\dfrac{1}{\text {Re} H^2} \left( \dfrac{\partial ^2 w}{\partial \xi ^2}+\dfrac{1}{\xi }\dfrac{\partial w}{\partial \xi } \right) \nonumber \\&\dfrac{1}{\text {Re}}\left[ \dfrac{\partial ^2 w}{\partial z^2}-\dfrac{2\xi }{H} \dfrac{dH}{dz}\dfrac{\partial ^2 w}{\partial \xi \partial z}-\dfrac{\xi }{H}\dfrac{d^2 H}{dz^2}\dfrac{\partial w}{\partial \xi }+\dfrac{\xi ^2}{H^2}\left( \dfrac{dH}{dz} \right) ^2\dfrac{\partial ^2 w}{\partial \xi ^2}+\dfrac{3\xi }{H^2} \left( \dfrac{dH}{dz} \right) ^2 \dfrac{\partial w}{\partial \xi } \right] \nonumber \\&-\dfrac{1}{\text {Re}}M^2w \end{aligned}$$

3.4 $$ \begin{aligned}   \frac{{\partial \theta }}{{\partial \tau }} =  &  - \frac{{u\partial \theta }}{{H\partial \xi }} - w\left( {\frac{{\partial \theta }}{{\partial z}} - \frac{\xi }{H}\frac{{dH}}{{dz}}\frac{{\partial \theta }}{{\partial \xi }}} \right) + \frac{1}{{{\text{Pr Re}}H^{2} }}\left( {\frac{{\partial ^{2} \theta }}{{\partial \xi ^{2} }} + \frac{1}{\xi }\frac{{\partial \theta }}{{\partial \xi }}} \right) + \frac{{{\text{Ec}}}}{{{\text{Re}}}}{\text{M}}^{2} w^{2}  +  \\     & \frac{1}{{{\text{Pr Re}}}}\left[ {\frac{{\partial ^{2} \theta }}{{\partial z^{2} }} - \frac{{2\xi }}{H}\frac{{dH}}{{dz}}\frac{{\partial ^{2} \theta }}{{\partial \xi \partial z}} - \frac{\xi }{H}\frac{{d^{2} H}}{{dz^{2} }}\frac{{\partial \theta }}{{\partial \xi }} + \frac{{\xi ^{2} }}{{H^{2} }}\left( {\frac{{dH}}{{dz}}} \right)^{2} \frac{{\partial ^{2} \theta }}{{\partial \xi ^{2} }} + \frac{{3\xi }}{{H^{2} }}\left( {\frac{{dH}}{{dz}}} \right)^{2} \frac{{\partial \theta }}{{\partial \xi }}} \right] +  \\     & \frac{{{\text{Ec}}}}{{{\text{Re}}}}\left[ {2\left( {\frac{1}{H}\frac{{\partial w}}{{\partial \xi }}} \right)^{2}  + 2\left( {\frac{{\partial u}}{{\partial z}} - \frac{\xi }{H}\frac{{\partial H}}{{\partial z}}\frac{{\partial u}}{{\partial \xi }}} \right)^{2}  + \left( {\frac{{\partial w}}{{\partial z}} - \frac{\xi }{H}\frac{{\partial H}}{{\partial z}}\frac{{\partial w}}{{\partial \xi }} + \frac{1}{H}\frac{{\partial u}}{{\partial \xi }}} \right)^{2} } \right] \\  \end{aligned}  $$

3.5 $$ \begin{aligned}   \frac{{\partial C}}{{\partial \tau }} =  &  - \frac{{u\partial C}}{{H\partial \xi }} - w\left( {\frac{{\partial C}}{{\partial z}} - \frac{\xi }{H}\frac{{dH}}{{dz}}\frac{{\partial C}}{{\partial \xi }}} \right) + \frac{1}{{{\text{Sc Re}}H^{2} }}\left( {\frac{{\partial ^{2} C}}{{\partial \xi ^{2} }} + \frac{1}{\xi }\frac{{\partial C}}{{\partial \xi }}} \right) + \frac{1}{{{\text{Re}}}}\beta C +  \\     & \frac{1}{{{\text{Sc Re}}}}\left[ {\frac{{\partial ^{2} C}}{{\partial z^{2} }} - \frac{{2\xi }}{H}\frac{{dH}}{{dz}}\frac{{\partial ^{2} C}}{{\partial \xi \partial z}} - \frac{\xi }{H}\frac{{d^{2} H}}{{dz^{2} }}\frac{{\partial C}}{{\partial \xi }} + \frac{{\xi ^{2} }}{{H^{2} }}\left( {\frac{{dH}}{{dz}}} \right)^{2} \frac{{\partial ^{2} C}}{{\partial \xi ^{2} }} + \frac{{3\xi }}{{H^{2} }}\left( {\frac{{dH}}{{dz}}} \right)^{2} \frac{{\partial C}}{{\partial \xi }}} \right] +  \\     & \frac{{{\text{Sr}}}}{{{\text{Re}}}}\left[ {\frac{{\partial ^{2} \theta }}{{\partial z^{2} }} - \frac{{2\xi }}{H}\frac{{dH}}{{dz}}\frac{{\partial ^{2} \theta }}{{\partial \xi \partial z}} - \frac{\xi }{H}\frac{{d^{2} H}}{{dz^{2} }}\frac{{\partial \theta }}{{\partial \xi }} + \frac{{\xi ^{2} }}{{H^{2} }}\left( {\frac{{dH}}{{dz}}} \right)^{2} \frac{{\partial ^{2} \theta }}{{\partial \xi ^{2} }} + \frac{{3\xi }}{{H^{2} }}\left( {\frac{{dH}}{{dz}}} \right)^{2} \frac{{\partial \theta }}{{\partial \xi }}} \right] +  \\     & \frac{{{\text{Sr}}}}{{{\text{Re}}H^{2} }}\left( {\frac{{\partial ^{2} \theta }}{{\partial \xi ^{2} }} + \frac{1}{\xi }\frac{{\partial \theta }}{{\partial \xi }}} \right) \\  \end{aligned}  $$

The model above is solved subject to the following conditions

3.6 $$\begin{aligned}&w(\xi , z, 0)=w_0,\quad \theta (\xi , z, 0)=\theta _0, \quad C(\xi , z, 0)=c_0 \end{aligned}$$

3.7 $$\begin{aligned}&w(\xi , z, \tau )=u(\xi , z, \tau )=0 \quad \theta (\xi , z, \tau )=\theta _w, \quad C(\xi , z, \tau )=c_w \quad \text {on} \quad \xi = 1 \end{aligned}$$

3.8 $$\begin{aligned}&\dfrac{\partial w(\xi , z, \tau )}{\partial \xi }=\dfrac{\partial \theta (\xi , z, \tau )}{\partial \xi }=\dfrac{\partial C(\xi , z, \tau )}{\partial \xi }=u(\xi , z, \tau )=0 \quad \text {on} \quad \xi =0 \end{aligned}$$

### The radial momentum

We now find the radial velocity in terms of of the axial momentum. I that regard, we multiply the continuity equation by $\xi H$ and integrate with respect to $\xi$ subject to the given boundary conditions. This gives

3.9 $$\begin{aligned} u(\xi , z, \tau )=\xi \dfrac{dH}{dz}w \end{aligned}$$

From the radial momentum above, we find the partial derivatives $\dfrac{\partial u}{\partial \xi }$ and $\dfrac{\partial u}{\partial z}$ using the chain rule. This gives $\dfrac{\partial u}{\partial \xi }=\dfrac{dH}{dz}\left( \xi \dfrac{\partial w}{\partial \xi }+w \right)$ and $\dfrac{\partial u}{\partial z}=\xi \left( \dfrac{dH}{dz} \dfrac{\partial w}{\partial z}+w\dfrac{d^2H}{dz^2}\right)$. These partial derivatives, eliminate the axial velocity in terms *u* as we now write it in terms of the axial velocity. Such elimination of axial velocity in terms *u*, gives the following equations;

3.10 $$\begin{aligned} \dfrac{\partial w}{\partial \tau }=&-w\left( \dfrac{\partial w}{\partial z}-\dfrac{\xi }{H}\dfrac{dH}{dz}\dfrac{\partial w}{\partial \xi }\right) +(A_{0}+A_{1}\cos (m\tau ))+\dfrac{1}{\text {Re} H^2} \left( \dfrac{\partial ^2 w}{\partial \xi ^2}+\dfrac{1}{\xi }\dfrac{\partial w}{\partial \xi } \right) \nonumber \\&\dfrac{1}{\text {Re}}\left[ \dfrac{\partial ^2 w}{\partial z^2}-\dfrac{2\xi }{H} \dfrac{dH}{dz}\dfrac{\partial ^2 w}{\partial \xi \partial z}-\dfrac{\xi }{H}\dfrac{d^2 H}{dz^2}\dfrac{\partial w}{\partial \xi }+\dfrac{\xi ^2}{H^2}\left( \dfrac{dH}{dz} \right) ^2\dfrac{\partial ^2 w}{\partial \xi ^2}+\dfrac{3\xi }{H^2} \left( \dfrac{dH}{dz} \right) ^2 \dfrac{\partial w}{\partial \xi } \right] \nonumber \\&-\dfrac{\xi w}{H} \dfrac{dH}{dz} \dfrac{\partial w}{\partial \xi } -\dfrac{1}{\text {Re}}M^2w \end{aligned}$$

3.11 $$ \begin{aligned}   \frac{{\partial \theta }}{{\partial \tau }} =  &  - \frac{{\xi w}}{H}\frac{{dH}}{{dz}}\frac{{\partial \theta }}{{\partial \xi }} - w\left( {\frac{{\partial \theta }}{{\partial z}} - \frac{\xi }{H}\frac{{dH}}{{dz}}\frac{{\partial \theta }}{{\partial \xi }}} \right) + \frac{1}{{{\text{Pr Re}}H^{2} }}\left( {\frac{{\partial ^{2} \theta }}{{\partial \xi ^{2} }} + \frac{1}{\xi }\frac{{\partial \theta }}{{\partial \xi }}} \right) + \frac{{{\text{Ec}}}}{{{\text{Re}}}}{\text{M}}^{2} w^{2}  +  \\     & \frac{1}{{{\text{Pr Re}}}}\left[ {\frac{{\partial ^{2} \theta }}{{\partial z^{2} }} - \frac{{2\xi }}{H}\frac{{dH}}{{dz}}\frac{{\partial ^{2} \theta }}{{\partial \xi \partial z}} - \frac{\xi }{H}\frac{{d^{2} H}}{{dz^{2} }}\frac{{\partial \theta }}{{\partial \xi }} + \frac{{\xi ^{2} }}{{H^{2} }}\left( {\frac{{dH}}{{dz}}} \right)^{2} \frac{{\partial ^{2} \theta }}{{\partial \xi ^{2} }} + \frac{{3\xi }}{{H^{2} }}\left( {\frac{{dH}}{{dz}}} \right)^{2} \frac{{\partial \theta }}{{\partial \xi }}} \right] +  \\     & 2\frac{{{\text{Ec}}}}{{{\text{Re}}}}\left[ {\xi \left( {\frac{{dR}}{{dz}}\frac{{\partial w}}{{\partial z}} + w\frac{{d^{2} R}}{{dz^{2} }}} \right) - \frac{\xi }{H}\frac{{dH}}{{dz}}\frac{{dH}}{{dz}}\left( {\xi \frac{{\partial w}}{{\partial \xi }} + w} \right)} \right]^{2}  + 2\frac{{{\text{Ec}}}}{{{\text{Re}}}}\left( {\frac{1}{H}\frac{{\partial w}}{{\partial \xi }}} \right)^{2}  +  \\     & \frac{{{\text{Ec}}}}{{{\text{Re}}}}\left[ {\left( {\frac{{\partial w}}{{\partial z}} - \frac{\xi }{H}\frac{{\partial H}}{{\partial z}}\frac{{\partial w}}{{\partial \xi }} + \frac{1}{H}\frac{{dH}}{{dz}}\left( {\xi \frac{{\partial w}}{{\partial \xi }} + w} \right)} \right)^{2} } \right] \\  \end{aligned}  $$

3.12 $$ \begin{aligned}   \frac{{\partial C}}{{\partial \tau }} =  &  - \frac{{\xi w}}{H}\frac{{dH}}{{dz}}\frac{{\partial C}}{{\partial \xi }} - w\left( {\frac{{\partial C}}{{\partial z}} - \frac{\xi }{H}\frac{{dH}}{{dz}}\frac{{\partial C}}{{\partial \xi }}} \right) + \frac{1}{{{\text{Sc Re}}H^{2} }}\left( {\frac{{\partial ^{2} C}}{{\partial \xi ^{2} }} + \frac{1}{\xi }\frac{{\partial C}}{{\partial \xi }}} \right) - \frac{1}{{{\text{Re}}}}\beta C +  \\     & \frac{1}{{{\text{Sc Re}}}}\left[ {\frac{{\partial ^{2} C}}{{\partial z^{2} }} - \frac{{2\xi }}{H}\frac{{dH}}{{dz}}\frac{{\partial ^{2} C}}{{\partial \xi \partial z}} - \frac{\xi }{H}\frac{{d^{2} H}}{{dz^{2} }}\frac{{\partial C}}{{\partial \xi }} + \frac{{\xi ^{2} }}{{H^{2} }}\left( {\frac{{dH}}{{dz}}} \right)^{2} \frac{{\partial ^{2} C}}{{\partial \xi ^{2} }} + \frac{{3\xi }}{{H^{2} }}\left( {\frac{{dH}}{{dz}}} \right)^{2} \frac{{\partial C}}{{\partial \xi }}} \right] +  \\     & \frac{{{\text{Sr}}}}{{{\text{Re}}}}\left[ {\frac{{\partial ^{2} \theta }}{{\partial z^{2} }} - \frac{{2\xi }}{H}\frac{{dH}}{{dz}}\frac{{\partial ^{2} \theta }}{{\partial \xi \partial z}} - \frac{\xi }{H}\frac{{d^{2} H}}{{dz^{2} }}\frac{{\partial \theta }}{{\partial \xi }} + \frac{{\xi ^{2} }}{{H^{2} }}\left( {\frac{{dH}}{{dz}}} \right)^{2} \frac{{\partial ^{2} \theta }}{{\partial \xi ^{2} }} + \frac{{3\xi }}{{H^{2} }}\left( {\frac{{dH}}{{dz}}} \right)^{2} \frac{{\partial \theta }}{{\partial \xi }}} \right] +  \\     & \frac{{{\text{Sr}}}}{{{\text{Re}}H^{2} }}\left( {\frac{{\partial ^{2} \theta }}{{\partial \xi ^{2} }} + \frac{1}{\xi }\frac{{\partial \theta }}{{\partial \xi }}} \right) \\  \end{aligned}  $$

### Numerical procedure

To study the dynamics of blood flow, heat and mass transfer in a stenosed artery, we make use of the finite difference approximations. Developing the scheme for finite difference approximations, we put in place the central difference approximation in discretizing the spatial derivatives and also use the explicit forward finite difference approximation to discretize the time derivative. The same was used by^28^. To maintain stability it was ensured that $0<\dfrac{\Delta \tau }{ (\Delta \xi )^2}\le 0.5$ In that regard therefore, we have the following;

3.13 $$\begin{aligned} \dfrac{\partial w}{\partial \xi }&=\dfrac{w^k_{i,j+1}-w^k_{i,j-1}}{2\Delta \xi }, \quad \dfrac{\partial ^2 w}{\partial \xi ^2}=\dfrac{w^k_{i,j+1}-2w^k_{i,j}+w^k_{i,j-1}}{(\Delta \xi )^2},\quad \dfrac{\partial w}{\partial \tau }=\dfrac{w^{k+1}_{i,j}-w^k_{i,j}}{\Delta \tau } \end{aligned}$$

3.14 $$\begin{aligned} \dfrac{\partial w}{\partial z}&=\dfrac{w^k_{i+1,j}-w^k_{i-1,j}}{2\Delta z}, \quad \dfrac{\partial ^2 w}{\partial z^2}=\dfrac{w^k_{i+1,j}-2w^k_{i,j}+w^k_{i-1,j}}{(\Delta z)^2} \end{aligned}$$

3.15 $$\begin{aligned} \dfrac{\partial \theta }{\partial \xi }&=\dfrac{\theta ^k_{i+1,j}-\theta ^k_{i-1,j}}{2\Delta \xi }, \quad \dfrac{\partial ^2 \theta }{\partial \theta ^2}=\dfrac{\theta ^k_{i+1,j}-2\theta ^k_{i,j}+\theta ^k_{i-1,j}}{(\Delta \xi )^2},\quad \quad \dfrac{\partial \theta }{\partial \tau }=\dfrac{\theta ^{k+1}_{i,j}-\theta ^k_{i,j}}{\Delta \tau } \end{aligned}$$

3.16 $$\begin{aligned} \dfrac{\partial \theta }{\partial z}&=\dfrac{\theta ^k_{i+1,j}-\theta ^k_{i-1,j}}{2\Delta z},\quad \dfrac{\partial ^2 \theta }{\partial z^2}=\dfrac{\theta ^k_{i+1,j}-2\theta ^k_{i,j}+\theta ^k_{i-1,j}}{(\Delta z)^2} \end{aligned}$$

3.17 $$\begin{aligned} \dfrac{\partial C}{\partial \xi }&=\dfrac{C^k_{i+1,j}-C^k_{i-1,j}}{2\Delta \xi }, \quad \dfrac{\partial ^2 C}{\partial \xi ^2}=\dfrac{C^k_{i+1,j}-2C^k_{i,j}+C^k_{i-1,j}}{(\Delta \xi )^2}, \quad \dfrac{\partial C}{\partial \tau }=\dfrac{C^{k+1}_{i,j}-C^k_{i,j}}{\Delta \tau } \end{aligned}$$

3.18 $$\begin{aligned} \dfrac{\partial C}{\partial z}&=\dfrac{C^k_{i+1,j}-C^k_{i-1,j}}{2\Delta z},\quad \dfrac{\partial ^2 C}{\partial z^2}=\dfrac{C^k_{i+1,j}-2C^k_{i,j}+C^k_{i-1,j}}{(\Delta z)^2} \end{aligned}$$

In this case, $\Delta \xi$ is the increment in radial direction, $\Delta z$ is the increment in axial direction and $\Delta \tau$ is the increment in time.

Besides, the discretization of *w*(*i*, *j*, *k*), $\theta (i,j,k)$ and *C*(*i*, *j*, *k*) is written as $w^k_{i,j}$, $\theta ^k_{i,j}$ and $C^k_{i,j}$ respectively. The following definitions are also considered;

3.19 $$\begin{aligned} \xi _{j}= & {} (j-1)\Delta \xi ;\quad j=1,2,3,..., N+1 \quad \text {where},\quad \xi _{N+1}=1 \end{aligned}$$

3.20 $$\begin{aligned} z_{i}= & {} (j-1)\Delta z;\quad i=1,2,3,..., M+1 \quad \quad \quad \quad \quad \quad \quad \quad \quad \end{aligned}$$

3.21 $$\begin{aligned} \tau _{k}= & {} (k-1)\Delta t;\quad k=1,2,3...\quad \quad \quad \quad \quad \quad \quad \quad \quad \quad \quad \end{aligned}$$

Substituting the finite difference schemes into our model equations we get the following;

Momentum equation:

3.22 $$\begin{aligned} w^k_{i,j+1}=&w^k_{i,j}-\Delta \tau \left[ \dfrac{\xi _{j}}{H_{i}} \left( \dfrac{dH}{dz} \right) _{i}\left( \dfrac{w^k_{i,j+1}-w^k_{i,j-1}}{2\Delta \xi } \right) \right] +(A_{0}+A_{1}\cos (m_{1}t))\nonumber \\ -&\Delta \tau w^k_{i,j}\left[ \dfrac{w^k_{i+1,j}-w^k_{i-1,j}}{2\Delta z}-\dfrac{\xi _{j}}{H_{i}} \left( \dfrac{dH}{dz} \right) _{i} \dfrac{w^k_{i,j+1}-w^k_{i,j-1}}{2\Delta \xi } \right] \nonumber \\ +&\dfrac{\Delta \tau }{\text {Re} H^2}\left[ \dfrac{w^k_{i,j+1}-2w^k_{i,j}+w^k_{i,j-1}}{(\Delta \xi )^2}+\dfrac{1}{\xi _{j}} \left( \dfrac{w^k_{i,j+1}-w^k_{i,j-1}}{2\Delta \xi } \right) \right] \nonumber \\ +&\dfrac{\Delta \tau }{\text {Re}}\left[ \dfrac{w^k_{i+1,j}-2w^k_{i,j}+w^k_{i-1,j}}{(\Delta z)^2} -\dfrac{2 \xi _{j}}{H_{i}} \left( \dfrac{dH}{dz} \right) _{i} \left( \dfrac{w^k_{i+1,j+1}-w^k_{i-1,j+1}-w^k_{i+1,j-1}+w^k_{i-1,j-1}}{4\Delta \xi \Delta z} \right) \right] \nonumber \\ -&\dfrac{\Delta \tau \xi _{j}}{\text {Re} H_{i}}\left( \dfrac{d^2 H}{dz^2 } \right) _{i}\left( \dfrac{w^k_{i,j+1}-w^k_{i,j-1}}{2\Delta \xi } \right) + \dfrac{\Delta \tau \xi ^2_{j}}{\text {Re} H^2_{i}}\left( \dfrac{d H}{dz }\right) ^2_{i}\left( \dfrac{w^k_{i,j+1}-2w^k_{i,j}+w^k_{i,j-1}}{(\Delta \xi )^2} \right) \nonumber \\ +&\dfrac{3\Delta \tau \xi _{j}}{\text {Re} H^2_{i}}\left( \dfrac{d H}{dz } \right) ^2_{i}\left( \dfrac{w^k_{i,j+1}-w^k_{i,j-1}}{2\Delta \xi } \right) -\frac{\Delta \tau }{\text {Re}}\text {M}^2 w^k_{i,j} \end{aligned}$$

Energy equation:

3.23 $$ \begin{aligned}   \theta _{{i,j}}^{{k + 1}}  =  & \theta _{{i,j}}^{k}  - \Delta \tau \left[ {\frac{{\xi _{j} }}{{H_{i} }}\left( {\frac{{dH}}{{dz}}} \right)_{i} \left( {w_{{i,j}}^{k} } \right)\left( {\frac{{\theta _{{i,j + 1}}^{k}  - \theta _{{i,j - 1}}^{k} }}{{2\Delta \xi }}} \right)} \right] \\     &  - \Delta \tau \left[ {w_{{i,j}}^{k} \left( {\frac{{\theta _{{i + 1,j}}^{k}  - \theta _{{i - 1,j}}^{k} }}{{2\Delta z}} - \frac{{\xi _{j} }}{{H_{i} }}\left( {\frac{{dH}}{{dz}}} \right)_{i} \left( {\frac{{\theta _{{i,j + 1}}^{k}  - \theta _{{i,j - 1}}^{k} }}{{2\Delta \xi }}} \right)} \right)} \right] \\     &  + \frac{{\Delta \tau }}{{{\text{Pr Re}}}}\left[ {\frac{{\theta _{{i,j + 1}}^{k}  - 2\theta _{{i,j}}^{k}  + \theta _{{i,j - 1}}^{k} }}{{H_{i}^{2} (\Delta \xi )^{2} }} + \frac{{\theta _{{i,j + 1}}^{k}  - \theta _{{i,j - 1}}^{k} }}{{2\xi _{j} H_{i}^{2} \Delta \xi }}} \right] + \frac{{{\text{Ec}}}}{{{\text{Re}}}}{\text{M}}^{2} w_{{i,j}}^{k}  \\     &  - \frac{{\Delta \tau }}{{{\text{Pr Re}}}}\left[ {\frac{{2\xi _{j} }}{{H_{i} }}\frac{{dH}}{{dz}}\left( {\frac{{\theta _{{i + 1,j + 1}}^{k}  - \theta _{{i - 1,j + 1}}^{k}  - \theta _{{i + 1,j - 1}}^{k}  + C_{{i - 1,j - 1}}^{k} }}{{4\Delta \xi \Delta z}}} \right)} \right] \\     &  - \frac{{\Delta \tau }}{{{\text{Pr Re}}}}\left[ {\frac{{\xi _{j} }}{{H_{i} }}\left( {\frac{{d^{2} H}}{{dz^{2} }}} \right)_{i} \left( {\frac{{\theta _{{i,j + 1}}^{k}  - \theta _{{i,j - 1}}^{k} }}{{2\Delta \xi }}} \right) + \left( {\frac{{\xi _{j} }}{{H_{i} }}} \right)^{2} \left( {\frac{{dH}}{{dz}}} \right)_{i}^{2} \left( {\frac{{\theta _{{i,j + 1}}^{k}  - 2\theta _{{i,j}}^{k}  + \theta _{{i,j - 1}}^{k} }}{{(\Delta \xi )^{2} }}} \right)} \right] \\     &  + \frac{{\Delta \tau }}{{{\text{Pr Re}}}}\left[ {\frac{{3\xi _{j} }}{{H_{i}^{2} }}\left( {\frac{{dH}}{{dz}}} \right)_{i}^{2} \left( {\frac{{\theta _{{i,j + 1}}^{k}  - \theta _{{i,j - 1}}^{k} }}{{2\Delta \xi }}} \right)} \right] \\     &  + \frac{{2\Delta \tau {\text{Ec}}}}{{{\text{Re}}}}\left[ {\xi _{j} \left( {\left( {\frac{{dH}}{{dz}}} \right)_{i} \left( {\frac{{w_{{i,j + 1}}^{k}  - w_{{i,j - 1}}^{k} }}{{2\Delta z}}} \right)} \right) + w_{{i,j}}^{k} \left( {\frac{{d^{2} H}}{{dz^{2} }}} \right)_{i}  - \frac{{\xi _{j} }}{{H_{i} }}\left( {\frac{{dH}}{{dz}}} \right)^{2} \left( {\xi _{j} \left( {\frac{{w_{{i,j + 1}}^{k}  - w_{{i,j - 1}}^{k} }}{{2\Delta \xi }}} \right) + w_{{i,j}}^{k} } \right)} \right]^{2}  \\     &  + \frac{{{\text{Ec}}\Delta \tau }}{{{\text{Re}}}}\left[ {\left( {\frac{{w_{{i,j + 1}}^{k}  - w_{{i,j - 1}}^{k} }}{{2\Delta z}}} \right) - \frac{\xi }{{H_{i} }}\frac{{dH}}{{dz}}\left( {\frac{{w_{{i,j + 1}}^{k}  - w_{{i,j - 1}}^{k} }}{{2\Delta \xi }}} \right) + \frac{1}{{H_{i} }}\frac{{dH}}{{dz}}\left( {\xi _{j} \left( {\frac{{w_{{i,j + 1}}^{k}  - w_{{i,j - 1}}^{k} }}{{2\Delta \xi }}} \right) + w_{{i,j}}^{k} } \right)} \right]^{2}  \\     &  + \frac{{2\Delta \tau {\text{Ec}}}}{{{\text{Re}}}}\left[ {\frac{1}{{H_{i} }}\left( {\frac{{w_{{i,j + 1}}^{k}  - w_{{i,j - 1}}^{k} }}{{2\Delta \xi }}} \right)} \right]^{2}  \\  \end{aligned}  $$

Concentration equation:

3.24 $$ \begin{aligned}   C_{{i,j}}^{{k + 1}}  =  & C_{{i,j}}^{k}  - \Delta \tau \left[ {\frac{{\xi _{j} }}{{H_{i} }}\left( {\frac{{dH}}{{dz}}} \right)_{i} \left( {w_{{i,j}}^{k} } \right)\left( {\frac{{C_{{i,j + 1}}^{k}  - C_{{i,j - 1}}^{k} }}{{2\Delta \xi }}} \right)} \right] \\     &  - \Delta \tau \left[ {w_{{i,j}}^{k} \left( {\frac{{C_{{i + 1,j}}^{k}  - C_{{i - 1,j}}^{k} }}{{2\Delta z}} - \frac{{\xi _{j} }}{{H_{i} }}\left( {\frac{{dH}}{{dz}}} \right)_{i} \left( {\frac{{C_{{i,j + 1}}^{k}  - C_{{i,j - 1}}^{k} }}{{2\Delta \xi }}} \right)} \right)} \right] \\     &  + \frac{{\Delta \tau }}{{{\text{Sc Re}}}}\left[ {\frac{{C_{{i,j + 1}}^{k}  - 2C_{{i,j}}^{k}  + C_{{i,j - 1}}^{k} }}{{H_{i}^{2} (\Delta \xi )^{2} }} + \frac{{C_{{i,j + 1}}^{k}  - C_{{i,j - 1}}^{k} }}{{2\xi _{j} H_{i}^{2} \Delta \xi }}} \right] \\     &  - \frac{{\Delta \tau }}{{{\text{Sc Re}}}}\left[ {\frac{{2\xi _{j} }}{{H_{i} }}\frac{{dH}}{{dz}}\left( {\frac{{C_{{i + 1,j + 1}}^{k}  - C_{{i - 1,j + 1}}^{k}  - C_{{i + 1,j - 1}}^{k}  + C_{{i - 1,j - 1}}^{k} }}{{4\Delta \xi \Delta z}}} \right)} \right] +  \\     &  - \frac{{\Delta \tau }}{{{\text{Sc Re}}}}\left[ {\frac{{\xi _{j} }}{{H_{i} }}\left( {\frac{{d^{2} H}}{{dz^{2} }}} \right)_{i} \left( {\frac{{C_{{i,j + 1}}^{k}  - C_{{i,j - 1}}^{k} }}{{2\Delta \xi }}} \right) + \left( {\frac{{\xi _{j} }}{{H_{i} }}} \right)^{2} \left( {\frac{{dH}}{{dz}}} \right)_{i}^{2} \left( {\frac{{C_{{i,j + 1}}^{k}  - 2C_{{i,j}}^{k}  + C_{{i,j - 1}}^{k} }}{{(\Delta \xi )^{2} }}} \right)} \right] \\     &  + \frac{{\Delta \tau }}{{{\text{Sc Re}}}}\left[ {\frac{{3\xi _{j} }}{{H_{i}^{2} }}\left( {\frac{{dH}}{{dz}}} \right)_{i}^{2} \left( {\frac{{C_{{i,j + 1}}^{k}  - C_{{i,j - 1}}^{k} }}{{2\Delta \xi }}} \right)} \right] \\     &  + \frac{{{\text{Sr}}}}{{{\text{Re}}}}\Delta \tau \left[ {\frac{{3\xi _{j} }}{{H_{i}^{2} }}\left( {\frac{{dH}}{{dz}}} \right)_{i}^{2} \left( {\frac{{\theta _{{i,j + 1}}^{k}  - \theta _{{i,j - 1}}^{k} }}{{2\Delta \xi }}} \right) + \left( {\frac{{\theta _{{i,j + 1}}^{k}  - 2\theta _{{i,j}}^{k}  + \theta _{{i,j - 1}}^{k} }}{{(\Delta z)^{2} }}} \right)} \right] \\     &  - \frac{{2\xi _{j} \Delta \tau }}{{H_{i} }}\frac{{{\text{Sr}}}}{{{\text{Re}}}}\left( {\frac{{dH}}{{dz}}} \right)_{i} \left( {\frac{{\theta _{{i + 1,j + 1}}^{k}  - \theta _{{i - 1,j + 1}}^{k}  - \theta _{{i + 1,j - 1}}^{k}  + \theta _{{i - 1,j - 1}}^{k} }}{{4\Delta \xi \Delta z}}} \right) \\  \end{aligned}  $$

3.25 $$ \begin{aligned}    - \frac{{{\text{Sr}}}}{{{\text{Re}}}}\Delta \tau \left[ {\frac{{\xi _{j} }}{{H_{i} }}\left( {\frac{{d^{2} H}}{{dz^{2} }}} \right)_{i} \left( {\frac{{\theta _{{i,j + 1}}^{k}  - \theta _{{i,j - 1}}^{k} }}{{2\Delta \xi }}} \right)} \right] + \Delta \tau \frac{{{\text{Sr}}}}{{{\text{Re}}}}\left( {\frac{{\xi _{j} }}{{H_{i} }}} \right)^{2} \left( {\frac{{dH}}{{dz}}} \right)_{i}^{2} \left( {\frac{{\theta _{{i,j + 1}}^{k}  - 2\theta _{{i,j}}^{k}  + \theta _{{i,j - 1}}^{k} }}{{(\Delta \xi )^{2} }}} \right) \\     &  + \Delta \tau \frac{{{\text{Sr}}}}{{{\text{Re}}H_{i}^{2} }}\left[ {\left( {\frac{{\theta _{{i,j + 1}}^{k}  - 2\theta _{{i,j}}^{k}  + \theta _{{i,j - 1}}^{k} }}{{(\Delta \xi )^{2} }}} \right) + \frac{1}{{\xi _{j} }}\left( {\frac{{\theta _{{i,j + 1}}^{k}  - \theta _{{i,j - 1}}^{k} }}{{2\Delta \xi }}} \right)} \right] - \frac{1}{{{\text{Re}}}}\beta C_{{i,j}}^{k}  \\  \end{aligned}  $$

We now discretize the boundary conditions. The Neumann boundary condition at $\xi =0$ is given as:

3.26 $$\begin{aligned} \dfrac{\partial w}{\partial \xi }=\dfrac{w^k_{i,j+1}-w^k_{i,j-1}}{2\Delta \xi }=\dfrac{\partial \theta }{\partial \xi }=\dfrac{\theta ^k_{i,j+1}-\theta ^k_{i,j-1}}{2\Delta \xi }=\dfrac{\partial C}{\partial \xi }=\dfrac{C^k_{i,j+1}-C^k_{i,j-1}}{2\Delta \xi }=0 \end{aligned}$$

The equation above leads to the relations $w^k_{i,j+1}-w^k_{i,j-1}=0$, $\theta ^k_{i,j+1}-\theta ^k_{i,j-1}=0$ and $C^k_{i,j+1}-C^k_{i,j-1}=0$ implying that $w^k_{i,j+1}=w^k_{i,j-1}$, $\theta ^k_{i,j+1}=\theta ^k_{i,j-1}$ and $C^k_{i,j+1}=C^k_{i,j-1}$. Thus, at $\xi =0$ implies that $j=1$ which eventually gives $w^k_{i,2}=w^k_{i,0}$, $\theta ^k_{i,2}=\theta ^k_{i,0}$ and $C^k_{i,2}=C^k_{i,0}$. Since $w^k_{i,0}$, $\theta ^k_{i,0}$ and $C^k_{i,0}$ are ghost points, the derivatives $\dfrac{\partial w}{\partial \xi } \Bigg |{}_{j=1}$, $\dfrac{\partial \theta }{\partial \xi } \Bigg |{}_{j=1}$ and $\dfrac{\partial C}{\partial \xi } \Bigg |{}_{j=1}$are approximated using the denominator $\Delta \xi$ as shown below.

3.27 $$\begin{aligned} \dfrac{\partial w}{\partial \xi } \Bigg |{}_{j=1}=\dfrac{w^k_{i,j+1}-w^k_{i,j}}{\Delta \xi }=0, \dfrac{\partial \theta }{\partial \xi } \Bigg |{}_{j=1}=\dfrac{\theta ^k_{i,j+1}-\theta ^k_{i,j}}{\Delta \xi }=0, \dfrac{\partial C}{\partial \xi } \Bigg |{}_{j=1}=\dfrac{C^k_{i,j+1}-C^k_{i,j}}{\Delta \xi }=0 \end{aligned}$$

which gives $w^k_{i,2}=w^k_{i,1}$, $\theta ^k_{i,2}=\theta ^k_{i,1}$ and $C^k_{i,2}=C^k_{i,1}$. Thus, the discretized boundary conditions are given as shown below.

3.28 $$\begin{aligned} w^k_{i,2}=w^k_{i,1}, \quad \theta ^k_{i,2}=\theta ^k_{i,1}, \quad C^k_{i,2}=C^k_{i,1} \quad \quad \theta ^1_{i,j}=\theta _{0},\quad w^1_{i,j}=W_{0}, \quad C^1_{i,j}=c_{0} \end{aligned}$$

the initial axial velocity $W_{0}=W(\xi )$ is given as

3.29 $$\begin{aligned} W_{0}=\left( \dfrac{A_{0}+A_{1}}{4}\right) \left( 1-(H\xi _{i})^2 \right) \end{aligned}$$

## Results and discussion

In the current section, the graphical simulations of velocity, temperature and concentration profiles are presented.

### Arterial blood’s velocity profiles

**Figure 2:**
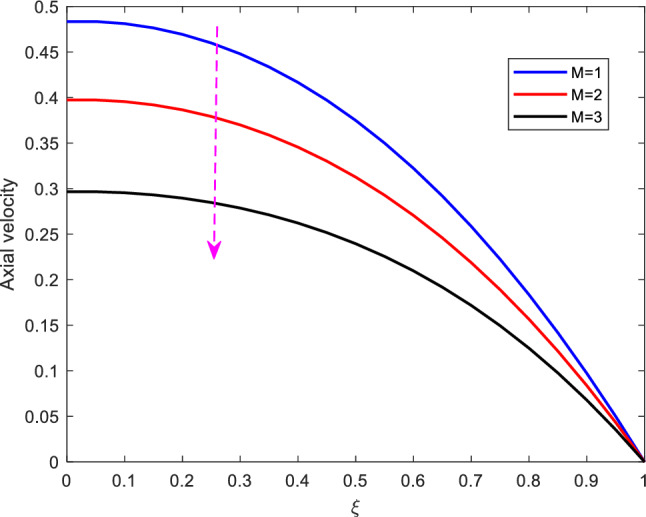
Effect of Hartman number on axial velocity.

Figure 2 shows the effect of Hartman number on the axial velocity. It is observed that as the Hartman number increases, the velocity profile diminishes. The Hartman number serves as a measure of the drag forces which result from the magnetic induction, the Lorentz force. This Lorentz force (which is in opposite direction) has the tendency of slowing down the motion. As the Hartman number increases, the Lorentz force increases and consequently diminishes the axial velocity. Besides, this physically implies that the velocity is diminished as a result of the enhancement of the increase in electrical conductivity. In that regard therefore, Magnetic fields can be used for clinical purposes to control the velocity of blood.

**Figure 3:**
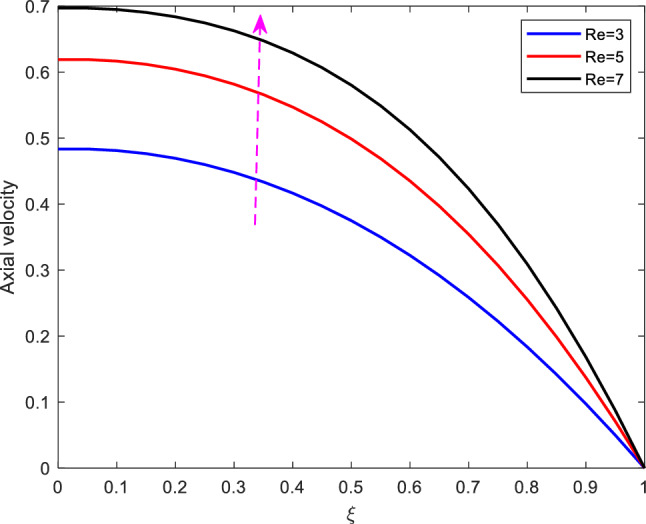
Effect of Reynolds number on axial velocity.

The effect of the Reynolds number on axial velocity is observed in Figure 3. From the figure, It is observed that increasing Reynolds number results to the increase in the axial velocity. The Reynolds number is the mathematical ratio of the inertial force to the viscous force present in the fluid (in this case blood). As Reynolds number increases the inertial force increases which results to the increase of the fluid’s velocity.

**Figure 4:**
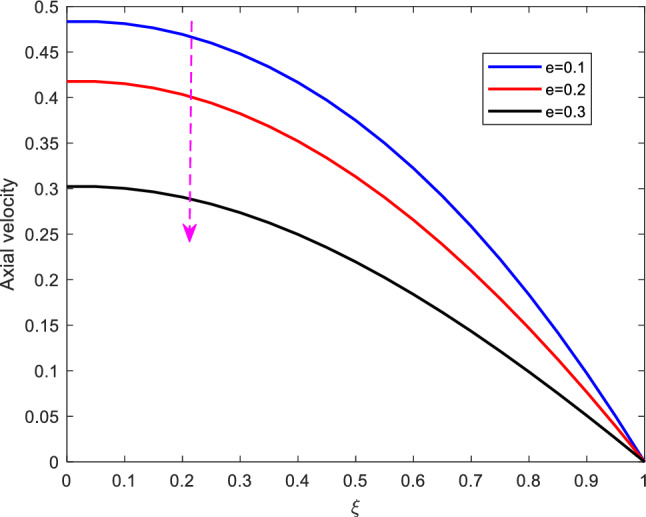
Effect of Stenotic height on axial velocity.

**Figure 5:**
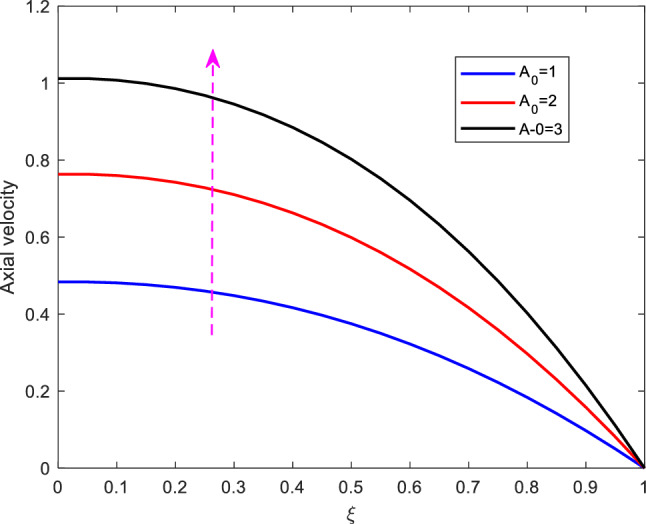
Effect of steady-state part of pressure gradient on axial velocity.

Figure 4 shows the effect of stenotic height on axial velocity. It is revealed that, as the stenotic height increases, the axial velocity declines. The decline of the axial velocity is reasoned in the manner that as the stenotic height increases, the flow region declines. Besides, the steady-state part of pressure gradient is varied to see its effect on axial velocity. as expected, increasing $A_0$ leads to the increase in the axial velocity profile. See Figure 5.

**Figure 6:**
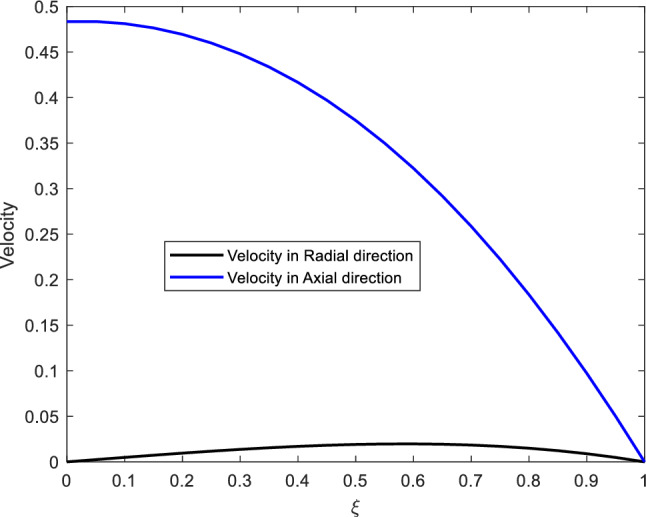
The arterial axial and radial velocities.

Figure 6 presents both, axial and radial velocities. We observe that, the radial velocity is much lesser than the axial velocity. This is because the study assumes that the pressure is more dominant in axial direction than in radial direction. The radial velocity is observed to start at velocity 0 and increases gradually, however ending with velocity 0 again on the arterial wall. this is to suit the no slip condition considered.

### Temperature profiles

This part presents the dynamics of temperature profiles for various parameters. the impact of increasing Prandtl number $\text {Pr}$, Eckert number $\text {Ec}$, Reynolds number $\text {Re}$ and the Hartman number $\text {M}$ is studied.

**Figure 7:**
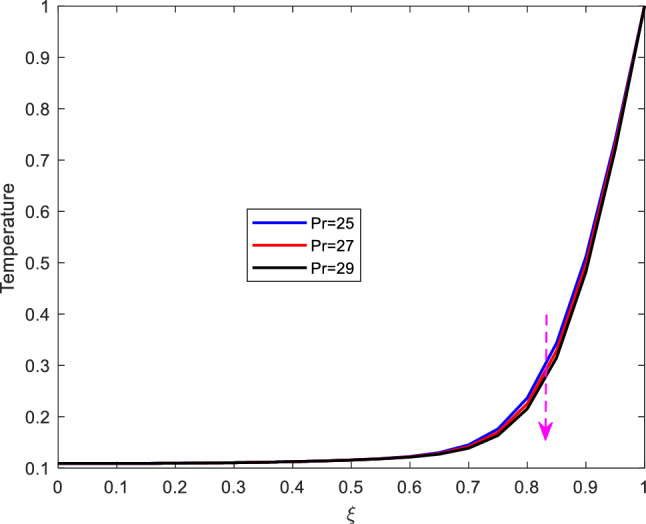
Effect of Prandtl number on temperature profile.

It is observed from Figure 7 that increase in Prandtl number, diminishes the temperature profiles. The Prandtl number represents the ratio of momentum diffusivity to thermal diffusivity. Increasing Prandtl number implies that the momentum diffusivity or kinematic viscosity is more dominant thermal diffusivity. Besides, increasing Prandtl number weakens the thermal boundary that results to reduction of the temperature profile.

**Figure 8:**
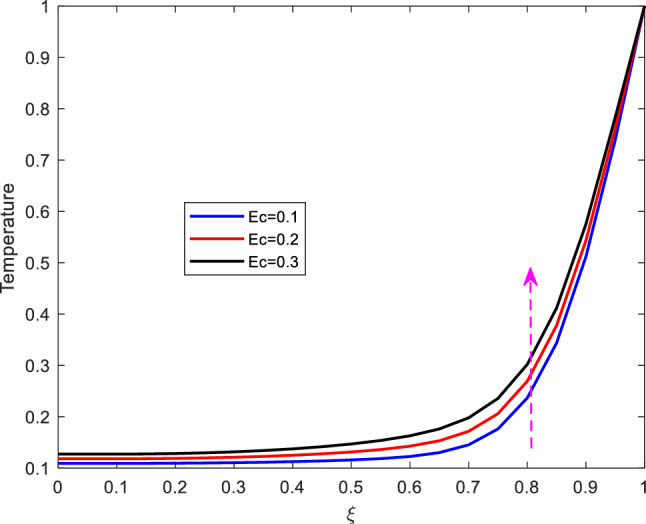
Effect of Eckert number on temperature profile.

The impact of altering Eckert number on temperature profile is shown on Figure 8. As shown from the figure, increase in Eckert number, enhances temperature. In this regard, the Eckert number characterizes the self heating of the fluid (blood) as a result of viscous dissipation. Increasing the Eckert number leads to increase in the internal friction of the fluid and eventually enhancing the fluid’s temperature profile. In this regard therefore, it is suggested that the effect of self heating of the fluid due to viscous dissipation should not be neglected.

**Figure 9:**
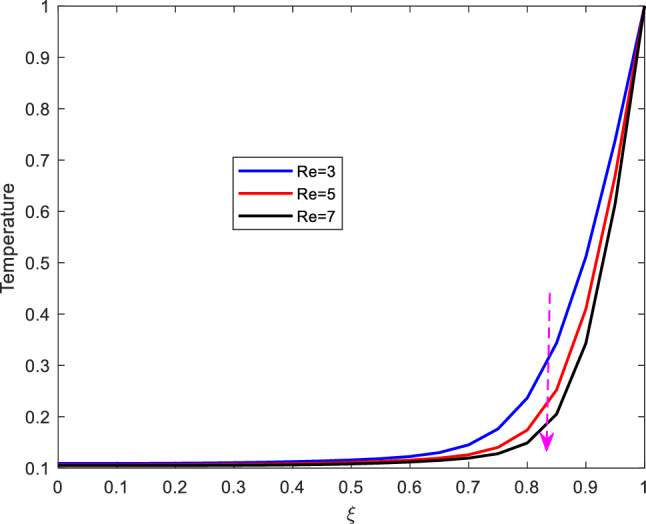
Effect of Reynolds number on temperature profile.

Figure 9 shows the effect of Reynolds number on temperature profile. From the figure, it is observed that the temperature declines with increase in the Reynolds number. The decline of the temperature is as a result of increase in inertial force. Besides, increase in the Reynolds number implies that inertial force is dominant than viscous force and thus diminishing heating which eventually declines the temperature.

**Figure 10:**
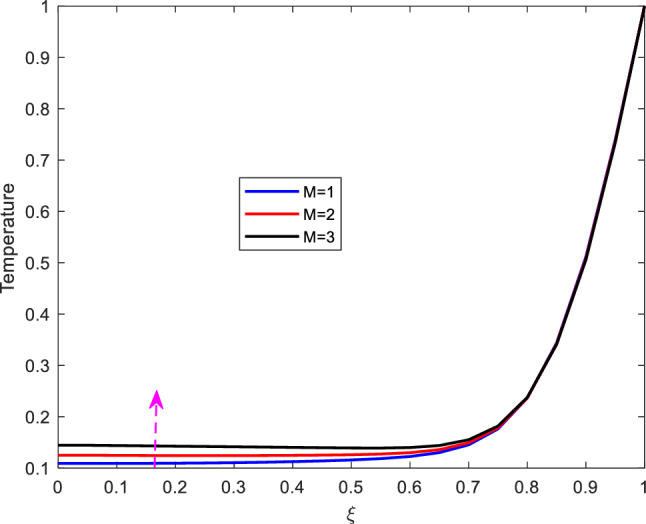
Effect of Hartman number on temperature profile.

The effect of Hartman number on temperature is shown on Figure 10. The figure reveals that, the increase in Hartman number, enhances temperature. Physically, increasing the Hartman number raises the characteristic value of te magnetic induction and the Lorentz force in general which results to increase in internal energy and the collision of electrons in the fluid, eventually enhancing the fluid’s temperature.

### Concentration profiles

The dynamics of concentration as a result of altering Schmidt number, Reynolds number, chemical reaction parameter, and Soret number is graphically presented.

**Figure 11:**
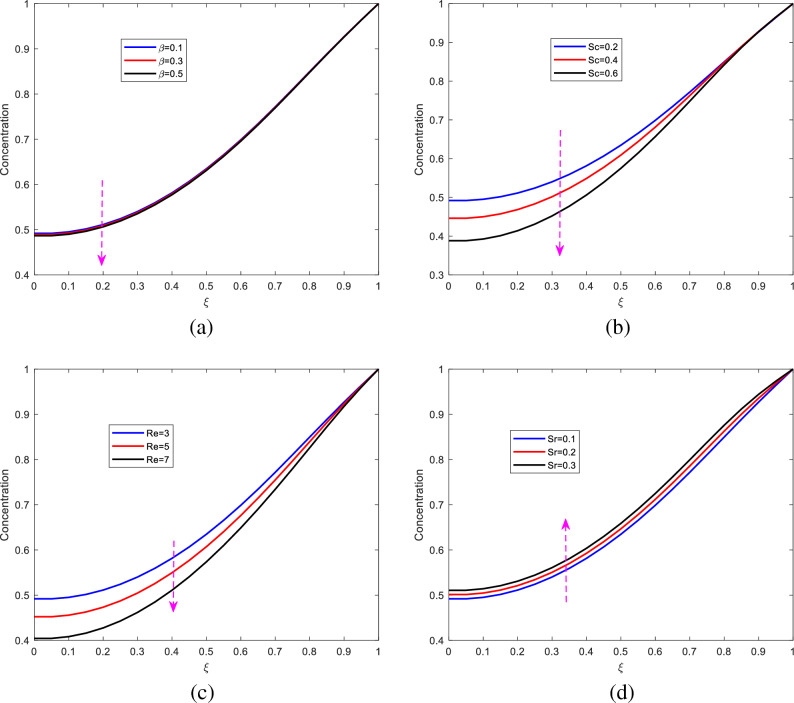
The Dynamics of the concentration profiles.

It is observed on Figure 11 that concentration declines with increase in chemical reaction, Schimdt number and the Reynolds number. However, concentration increases with Soret number. Chemical reaction therefore is like a destructive agent in the fluid. In the presence reaction, mass transfer diminishes. The concentration is also observed to decrease with increase in the Schmidt number. The Schmidt number is the ratio of the momentum diffusion to the mass diffusion. Increase in Schmidt number implies that the mass diffusion is dominated by the momentum or kinematic viscosity. As mass diffusion is dominated, the concentration declines. Regarding Reynolds number, its increase implies that the inertial force is dominant that the viscous force. Reynolds number enhances the flow velocity of the fluid, thus diminishing the concentration of the fluid due to the increased velocity.

Opposite behavior of the concentration is observed when the Soret number is altered. The concentration of the fluid increases with increase in the Soret number. Principally, the increase in Soret number, raises the temperature which results to high convective flow, this in turn increases the concentration.

## Comparison of the findings

**Figure 12:**
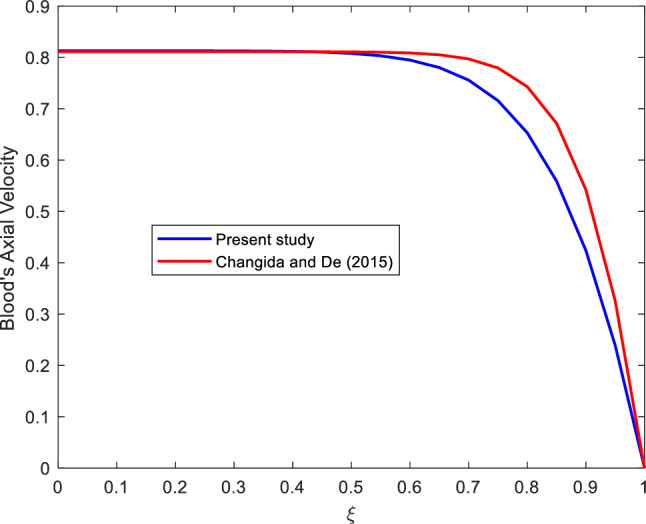
Model validation.

The results of the current study were compared with the previous similar study done by^29^ This is as shown in the Figure 12. In this regard, the velocity profile of the current study was plotted in same figure with that of^29^. Magnetic field for the current study was set to be zero. The profiles show a convincing agreement of the two studies though the previous study show involved the multiple stenosis of the artery.

## Conclusion

Modeling and computation of a constricted arterial blood flow, heat and mass transfer for a human body subjected to the magnetic resonance imaging (MRI) scan machine are studied. The simultaneous effects of magnetic fields, joule heating and chemical reaction have been investigated. The finite difference method was used to tackle the problem numerically. The simulation of fluids blood’s velocity, temperature and the concentration was done using the MATLAB software.

The study reveals that, the blood’s axial velocity diminishes with magnetic fields available in MRI scanning machine, height of stenosis and is enhanced with the Reynolds number and the steady-state part of the pressure gradient. An interesting finding is that, the axial velocity diminishes with stenotic height but the radial velocity increases with increase in height of stenosis.Temperature profile diminishes with increase in Prandtl number and Reynolds number.Opposite behavior is observed when Eckert number and Hartman number increase. Finally, The concentration profile declines with increase in chemical reaction parameter, Schmidt number and Reynolds number. Soret number is observed to enhance concentration.

## Data Availability

The datasets used and/or analyzed during the current study available from the corresponding author on reasonable request.

## List of symbols

*u*: Blood’s radial velocity
*w*: Blood’s axial velocity
*r*: Radial distance
*z*: Axial distance
$r_0$: Radius of the normal artery
*H*: Radius of the diseased artery
*e*: Protuberance
$z_0$: Half of the axial distance from the middle of stenosis
$\rho$: Density of the fluid
*t*: Dimensional time
$$\mu$$: Dynamic viscosity
$A_0$: Steady state part of pressure gradient
$A_1$: Amplitude of oscillatory
*n*: Heart pulse frequency
$$\sigma$$: Electrical conductivity
$$B_0$$: Magnetic strength
*C*: Mass concentration
$$C_0$$: Constant concentration
*D*: Diffusion coefficient
$\beta$: reaction rate (first order)
$c_p$: Specific heat capacity
*k*: Thermal conductivity
$K_T$: Thermal diffusion ratio
$\eta$: Dimensionless radial distance
$$\tau$$: Dimensionless time
$\xi$: Radial coordinate transformation variable
